# 1708. Molecular Epidemiology of *Staphylococcus aureus* Isolates Colonizing Infants in a Neonatal Intensive Care Unit: A 4-Year Cohort Study

**DOI:** 10.1093/ofid/ofad500.1541

**Published:** 2023-11-27

**Authors:** Christopher Reis, Leah M Siple, Raymond Stetson, Christopher A Collura, Jennifer Fang, Maria C Gallup, Tami Omdahl, Matthew Daniels, Jordan Starkey, Jean Barth, Priya Sampathkumar, Jun Chen, Robin Patel, W Charles Huskins

**Affiliations:** Mayo Clinic, Rochester, Minnesota; Mayo Clinic, Rochester, Minnesota; Mayo Clinic, Rochester, Minnesota; Mayo Clinic, Rochester, Minnesota; Mayo Clinic, Rochester, Minnesota; Mayo Clinic, Rochester, Minnesota; Mayo Clinic, Rochester, Minnesota; Mayo Clinic, Rochester, Minnesota; Mayo Clinic, Rochester, Minnesota; Mayo Clinic, Rochester, Minnesota; Mayo Clinic, Rochester, Minnesota; Mayo Clinic, Rochester, Minnesota; Mayo Clinic, Rochester, Minnesota; Mayo Clinic, Rochester, Minnesota

## Abstract

**Background:**

*Staphylococcus aureus* (SA) infections, both methicillin-susceptible and methicillin-resistant (MSSA, MRSA), cause significant morbidity among infants in neonatal intensive care units (NICU). Colonization usually precedes infection. We studied the molecular epidemiology of SA colonization in NICU infants using whole genome sequencing (WGS) and in relation to the opening of a new NICU with single-family rooms.

**Methods:**

From 2019-22, a prospective cohort of infants admitted to the Level IV NICU at Mayo Clinic, Rochester, MN was screened twice weekly for SA colonization using cultures of nares/axilla/groin swabs. Infection control procedures were consistent throughout, including use of Contact Precautions for infants colonized with MRSA but not for MSSA. The study was divided into period 1 (1/1/2019-11/30/2020) and period 2 (12/1/2020-12/31/2022) due to a move from a 34-bed unit (26 beds in four open-bay rooms and 8 beds in single-family rooms) to a new 34-bed unit (all single-family rooms). WGS was performed using Illumina MiSeq instrumentation and chemistry with Illumina Nextera XT library chemistry. Assembly and core genome multilocus sequence typing analysis were performed with Ridom SeqSphere+ software. Groups of related isolates were identified based on the number of allelic differences (related 0-8 differences, possibly related 9-29 differences, unrelated ≥ 30 differences). Groups of MRSA-related isolates were compared with reference USA genotypes. Chi square tests did not adjust for clustering.

**Results:**

There were 663 admissions in period 1 and 730 in period 2. The prevalence of MSSA and MRSA colonization was unchanged in the two periods (MSSA, 18.6% vs. 16.7%, p=0.37; MRSA 1.8% vs. 3.3%, p=0.08). By WGS, most MSSA and MRSA isolates were unrelated to another isolate in both periods (Table). Groups of related MSSA and MRSA isolates differed between the two periods and the number of isolates/group was small (< 6 isolates). Groups of related MRSA isolates were associated with reference genotypes USA100 (period 1), USA1000 (periods 1 & 2), and USA300 (period 2).

WGS Results for MSSA and MRSA in Periods 1 and 2

**Figure:**
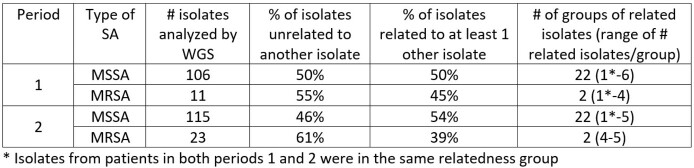

**Conclusion:**

NICU infants were usually colonized with unrelated strains of MSSA and MRSA, with only small clusters of related strains. The new NICU was not associated with a change in MSSA or MRSA colonization.

**Disclosures:**

**Priya Sampathkumar, MD**, Merck Vaccines: Advisor/Consultant **Robin Patel, MD**, Abbott Laboratories: Advisor/Consultant|Adaptive Phage Therapeutics: Grant/Research Support|Adaptive Phage Therapeutics: Mayo Clinic has a royalty-bearing know-how agreement and equity in Adaptive Phage Therapeutics.|BIOFIRE: Grant/Research Support|CARB-X: Advisor/Consultant|ContraFect: Grant/Research Support|Day Zero Diagnostics: Advisor/Consultant|HealthTrackRx: Advisor/Consultant|Mammoth Biosciences: Advisor/Consultant|Netflix: Advisor/Consultant|Oxford Nanopore Technologies: Advisor/Consultant|PhAST: Advisor/Consultant|See details: Patent on Bordetella pertussis/parapertussis PCR issued, a patent on a device/method for sonication with royalties paid by Samsung to Mayo Clinic|See details: continued, patent on an anti-biofilm substance issued|TenNor Therapeutics Limited: Grant/Research Support|Torus Biosystems: Advisor/Consultant|Trellis Bioscience, Inc.: Advisor/Consultant **W. Charles Huskins, MD, MSc**, ADMA Biologics: Advisor/Consultant|Bristol Myers Squibb: Stocks/Bonds|Pfizer: Advisor/Consultant|Pfizer: Stocks/Bonds|Zimmer Biomet: Stocks/Bonds

